# Supplementary material for: PDBspheres: a method for finding 3D similarities in local regions in proteins
Source: NAR Genom Bioinform. 2022 Oct 10;4(4):lqac078. doi: 10.1093/nargab/lqac078 (PMC9549786; doi:10.1093/nargab/lqac078)
Supplement: lqac078_Supplemental_Files [file lqac078_supplemental_files.zip › Supplementary Material.docx]

**File1: PDBspheres.COVID19_PL2pro.protein_ligand_summary.txt**


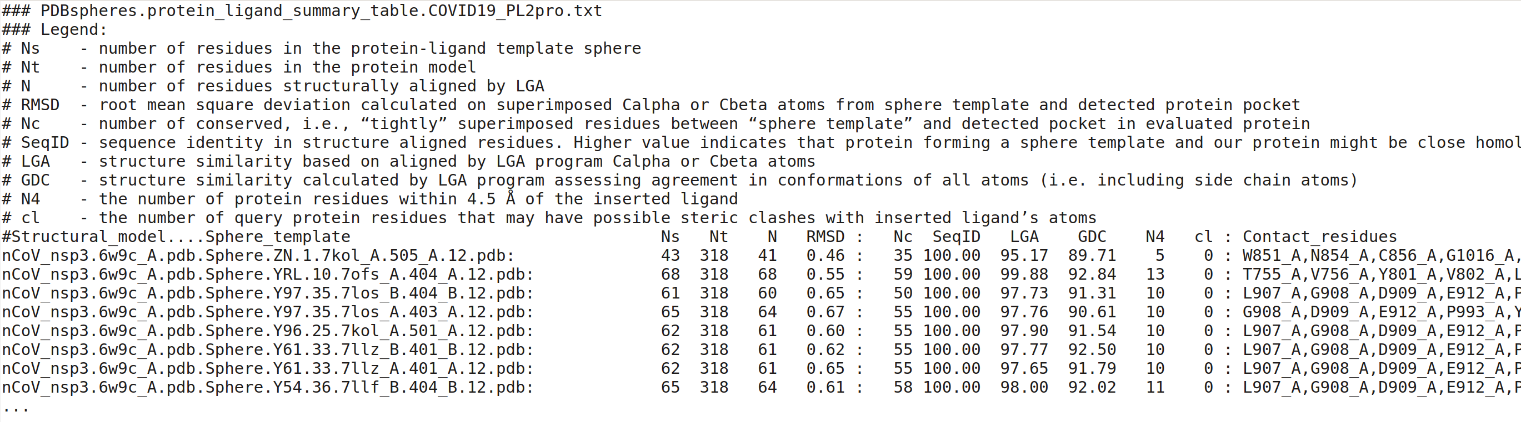


**File2: PDBspheres.PDBbind_Clusters.EC_included.txt**


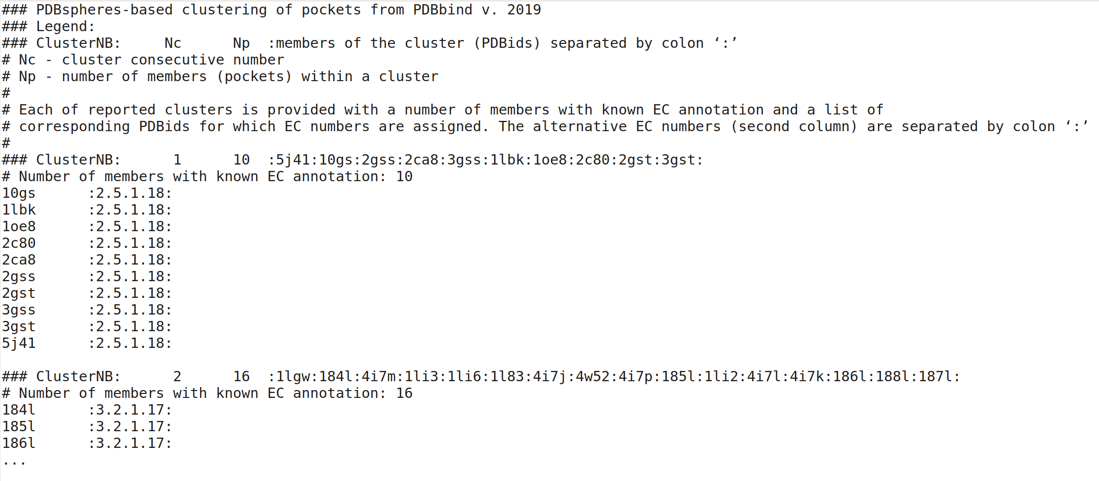


**File3: PDBspheres.PDBbind_Clusters.SCOP_included.txt**

(the same format as File2, but reporting SCOP annotation)

**File4: PDBspheres.PDBbind_Clusters.GO_included.txt**

(the same format as File2, but reporting GO annotation)

**File5: PDBspheres.PDBbind_Binding_sites_similarities.GDC_and_affinities.txt**


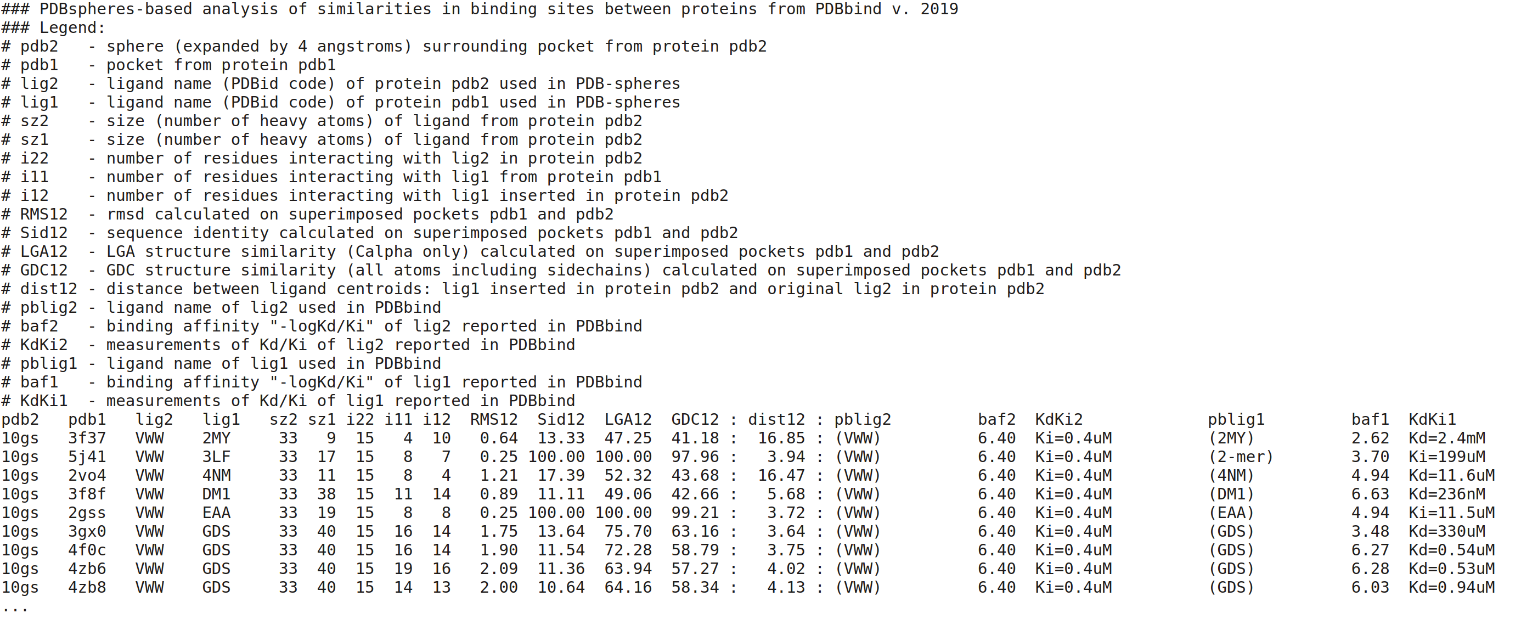


**File6: PDBspheres.PDBbind_Clusters.interactive_plot.html**

**(**interactive overview of predicted clusters shown on Figure.7.)
